# Detection of relapsing fever *Borrelia* spp., *Bartonella* spp. and Anaplasmataceae bacteria in argasid ticks in Algeria

**DOI:** 10.1371/journal.pntd.0006064

**Published:** 2017-11-16

**Authors:** Ismail Lafri, Basma El Hamzaoui, Idir Bitam, Hamza Leulmi, Reda Lalout, Oleg Mediannikov, Mohamed Chergui, Mohamed Karakellah, Didier Raoult, Philippe Parola

**Affiliations:** 1 Unité de Recherche en Maladies Infectieuses et Tropicales Emergentes (URMITE), Aix Marseille Université, UM63, CNRS 7278, IRD 198, Inserm 1095, AP-HM, Institut Hospitalo-Universitaire Méditerranée Infection, Marseille, France; 2 Institut des Sciences Vétérinaires, Université Blida 1, Blida, Algeria; 3 Université de Bab Ezzouar, Laboratoire d’Ecologie et Environnement, Algiers, Algeria; 4 Ecole Supérieure en Sciences de l'Aliment et des Industries Agroalimentaires (ESSAIA), El Harrach, Algiers, Algeria; 5 Ecole Nationale Vétérinaire de Toulouse, 23 chemin des capelles Toulouse, France; 6 EPH de Sidi Ali. Mostaganem. Ministère de la santé, de la population et de la réforme hospitalière, Mostaganem, Algeria; Institut Pasteur, FRANCE

## Abstract

**Background:**

Argasid ticks (soft ticks) are blood-feeding arthropods that can parasitize rodents, birds, humans, livestock and companion animals. Ticks of the *Ornithodoros* genus are known to be vectors of relapsing fever borreliosis in humans. In Algeria, little is known about relapsing fever borreliosis and other bacterial pathogens transmitted by argasid ticks.

**Methodology/Principal findings:**

Between May 2013 and October 2015, we investigated the presence of soft ticks in 20 rodent burrows, 10 yellow-legged gull (*Larus michahellis*) nests and animal shelters in six locations in two different bioclimatic zones in Algeria. Six species of argasid ticks were identified morphologically and through 16S rRNA gene sequencing. The presence and prevalence of *Borrelia* spp., *Bartonella* spp., *Rickettsia* spp. and Anaplasmataceae was assessed by qPCR template assays in each specimen. All qPCR-positive samples were confirmed by standard PCR, followed by sequencing the amplified fragments. Two *Borrelia* species were identified: *Borrelia hispanica* in *Ornithodoros occidentalis* in Mostaganem, and *Borrelia cf*. *turicatae* in *Carios capensis* in Algiers. One new *Bartonella* genotype and one new *Anaplasmataceae* genotype were also identified in *Argas persicus*.

**Conclusions:**

The present study highlights the presence of relapsing fever borreliosis agents, although this disease is rarely diagnosed in Algeria. Other bacteria of unknown pathogenicity detected in argasid ticks which may bite humans deserve further investigation.

## Author summary

Argasid ticks (soft ticks) are obligate blood-feeding arthropods that may bite humans. To increase understanding of the bacteria that are associated with these arthropods, we collected soft ticks from different sites in Algeria and used molecular tools to detect and identify bacteria in these ticks. Molecular tools revealed that some argasid ticks tested positive for agents of relapsing fever in humans, and other bacteria of unknown pathogenicity.

## Introduction

Ticks are obligatory hematophagous ectoparasites that can be vectors of protozoa, viruses and bacteria during their feeding process on animal hosts. They are currently considered to be second only to mosquitoes as vectors of human infectious disease around the world [1]. Two families of ticks are of medical significance: Ixodidae (hard ticks) and Argasidae (soft ticks). Hard ticks are the main ticks acting as vectors of human disease, but soft ticks are also known to transmit agents of human infectious diseases which are often neglected [2]. Argasid ticks comprise four genera and about 185 species, including three genera represented by a large range of species: *Carios* (87 species), *Argas* (57 species) and *Ornithodoros* (36 species). The fourth genus (*Otobius*) is represented by three species. This classification and the taxonomy of Argasids may evolve with the use of molecular tools [3]. Argasid ticks of the genus *Ornithodoros* include vectors of relapsing fever caused by *Borrelia* spp. in humans [4]. All these tick vectors are, for the most part, geographically restricted and are considered to be specific vectors of a given *Borrelia* spp. As *Borrelia* spp. may persist for many years in their long-lived vectors, *Ornithodoros* spp. are considered as both vectors and de facto reservoirs [2]. Vertebrate reservoirs of tick-borne relapsing fever (TBRF), *Borrelia* spp. include a variety of mammals, mainly rodents and insectivores, which inhabit burrows, dens and caves [5]. In Africa, TBRF *Borrelia* spp. include neglected vector-borne pathogens responsible for various febrile presentations and are most commonly suspected in malaria-like symptoms [6]. Tick-borne relapsing fever has been recognized as major cause of disease and death in several regions of Africa [7]. Currently, two agents of tick-borne relapsing fever have been detected in North Africa, namely *Borrelia crocidurae* and *Borrelia hispanica* [8]. An uncultured bacterium, “*Borrelia merionesi*,*”* has also been detected in *Ornithodoros* ticks in Morocco [9], and “*Candidatus* Borrelia algerica” has been reported in febrile patients in Algeria [10]. In Algeria, since the first human case of TBRF reported by Sergent in 1908 [11], the disease has been largely neglected, and recent epidemiological data are lacking. The local transmission of TBRF is not known, and cases are not diagnosed. In addition to “*Candidatus Borrelia algerica”* which has been detected in Oran, *Borrelia crocidurae* has been detected in *O*. *sonrai* (2.5% prevalence) [12]. No other bacteria are known to be associated with soft ticks in this area.

In this paper, we report a series of entomological investigations that we conducted between 2012 and 2015. We aimed to describe the distribution of soft ticks in Algeria and the prevalence of associated *Borrelia*, *Bartonella*, *Rickettsia* and *Anaplasma*.

## Materials and methods

### Ethical considerations

The study protocol was approved by the Steering Committee of the Algerian Ministry of Health (Direction Générale de la Prevention). Tick collection excluded national parks and protected areas and did not involve endangered or protected species (CITES, IUCN and national guidelines). All tick collections which took place inside homes and on private land were conducted after receiving permission from the owner.

### Tick sampling

This study was carried out between May 2012 and October 2015 in six locations in two different bioclimatic zones in Algeria. We conducted several sampling series in three northern coastal areas with a Mediterranean climate (Mostaganem, Algiers and El Tarf) and one in the highlands with a semi-arid climate (M’sila).

In Mostaganem, on the western coast of Algeria, three sites were investigated, including the port of Mostaganem (35°53’39” N, 0°05’25” E), Sidi Ali (36° 06’ 00” N, 0°25’00” E) and Achaacha (36° 14’ 47” N, 0°38’03” E). In Achaacha in March 2015Achaacha, we also specifically investigated rodent burrows within a farm. The owner of the farm had been hospitalized with an unexplained fever the previous year. Argasid ticks were also collected in Algiers from the central coastal area (the island of Agueli) (36° 44’ 00” N, 3°21’00” E), El Tarf (36° 42’ 2” N, 8°18’50” E) and M’sila in the eastern highlands of Algeria (35° 35’ 13” N, 4°40’08” E).

Ticks were sampled from a variety of natural and human-impacted habitats. In this study, we prospected rodent burrows and yellow-legged gull (*Larus michahellis*) nests, and inspected other animal shelters. We introduced a flexible tube into rodent burrows and aspirated the contents using a portable, petrol-powered aspirator [12]. Ticks from seabird nests were collected after the nesting period; the nests were recovered when the chicks had left their nests. The seabird nests were collected from between the rookeries on the island of Agueli (Algiers). The nests were then placed in individual bags to avoid parasite loss. Ticks were collected by sifting through the contents. Ticks were collected from slits inside animal shelters using entomological forceps. After collection, all biological materials were immediately stored in ethanol or liquid nitrogen (one tube per positive burrow/nest/animal shelter) and then forwarded to Marseille, France.

### Identification of ticks and the molecular detection of bacteria

#### Identification and molecular detection

Ticks were identified by morphological criteria using standard taxonomic keys [13]. Each specimen was rinsed twice in distilled water for 10 minutes and then dried on sterile filter paper. Handling was performed in a laminar flow biosafety cabinet. Ticks were individually crushed in sterile Eppendorf tubes. Total DNA was extracted in a final volume of 200 μl from one half of each ectoparasite using the QIAamp Tissue Kit (Qiagen, Hilden, Germany) by Qiagen-BioRobot EZ1 according to the manufacturer’s instructions [14]. Genomic DNA was stored at -20°C under sterile conditions. Once the DNA had been extracted, ticks were also identified by 16S rRNA gene sequencing, as previously described [12]. The DNA of each specimen was then used in qPCR template assays to detect *Bartonella* spp., *Rickettsia* spp., *Anaplasmataceae* bacteria and *Borrelia* spp. [15]. Negative controls were used in each qPCR and consisted of DNA extracted from uninfected ticks from our laboratory colony. Positive controls included DNA extracted from a dilution of cultured strains of *Borrelia crocidurae* (for the detection of *Borrelia* spp.), *Bartonella henselae* (for the detection of *Bartonella* spp.), *Rickettsia montanensis* (for the detection of *Rickettsia* spp.) and *Ehrlichia canis* (for the detection of Anaplasmataceae bacteria). Results were deemed positive if the Cycle threshold (Ct) value obtained by CFX96 was lower than 36. All samples identified as positive by qPCR (16S rDNA-based) for *Borrelia* spp. were confirmed by a standard PCR and sequencing for the fragments of the flagellin gene (*flaB*) [10]. For the detection of *Bartonella* spp., we targeted the 16S/23S RNA intergenic spacer (*ITS*) gene for qPCR [16] and confirmed the partial sequence of the citrate synthase gene (*gltA*) and the cell division protein gene (*ftsZ*) by sequencing. For the detection of *Rickettsia* spp., we targeted a partial sequence of the citrate synthase (*gltA*) (RKND03 system; *Rickettsia* genus-specific) for qPCR. Positive samples were tested by standard PCR targeting the *gltA* [17]. For detection of Anaplasmataceae bacteria, we targeted the 23S rRNA gene, as described [18] (Table 1). DNA sequencing reactions were performed on highly positive samples (Ct <28).

**Table 1:** Detection and identification of *Borrelia* spp., *Bartonella* spp. and Anaplasmataceae in Algerian argasid ticks.

Tick species (Genbank accession no. of molecular confirmation)	Nature of habitat	Location	Number of positive ticks by qPCR / total examined (% infected ticks)	Genotypes obtained by sequencing (Genbank accession number)
*Carios capensis* KP776644	Seabird nests	Algiers	5/48 (10.4%)	*Borrelia cf*. *turicatae*. *flaB* (MF432464)
*Ornithodoros occidentalis* KC311536	Rodent burrows	Achaacha (Mostaganem)	2/6 (33.3%)	*Borrelia hispanica*. *flaB* (MF432465)
*Argas persicus* GU451248	Animal shelters	Sidi Ali (Mostaganem)	4/50 (8%)	*Bartonella* AP1 Algeria *ftsZ* (MF432452)
				*Bartonella* AP2 Algeria *ftsZ* (MF432453)
				*Bartonella* AP3 Algeria *ftsZ* (MF432454)
				*Bartonella* AP4 Algeria *ftsZ* (MF432455)
				*Bartonella* AP1 Algeria *gltA* (MF432456)
				*Bartonella* AP2 Algeria *gltA* (MF432457)
			31/50 (62%)	*Anaplasma* AP5 Algeria *23S* (MF432458)
				*Anaplasma* AP8 Algeria *23S* (MF432459)
				*Anaplasma* AP10 Algeria *23S* (MF432460)
				*Anaplasma* AP11 Algeria *23S* (MF432461)
				*Anaplasma* AP14 Algeria *23S* (MF432462)
				*Anaplasma* AP15 Algeria *23S* (MF432463)
*Ornithodoros rupestris* KC311545	Rodent burrows	Mostaganem (Port)	0/34	-
*Ornithodoros sonrai* KC311525	Rodent burrows	M’sila	0/8	-
*Ornithodoros erraticus* KC311540	Rodent burrows	El Tarf	0/58	-

### Phylogenetic analysis

Phylogenetic trees were drawn using the neighbor-joining method from an alignment of the different genes used in the experiments. Sequences were aligned using CLUSTALW, and phylogenetic inferences obtained using the ML phylogenetic analysis with the TOPALi 2.5 software (Biomathematics and Statistics Scotland, Edinburgh, UK) within the integrated ML application, using the K81uf + I + Г substitution model. Numbers at the nodes are percentages of bootstrap values obtained by repeating the analysis from 100 replicates to generate a majority consensus tree.

## Results

### Sample collection and tick identification

**Fig 1:**
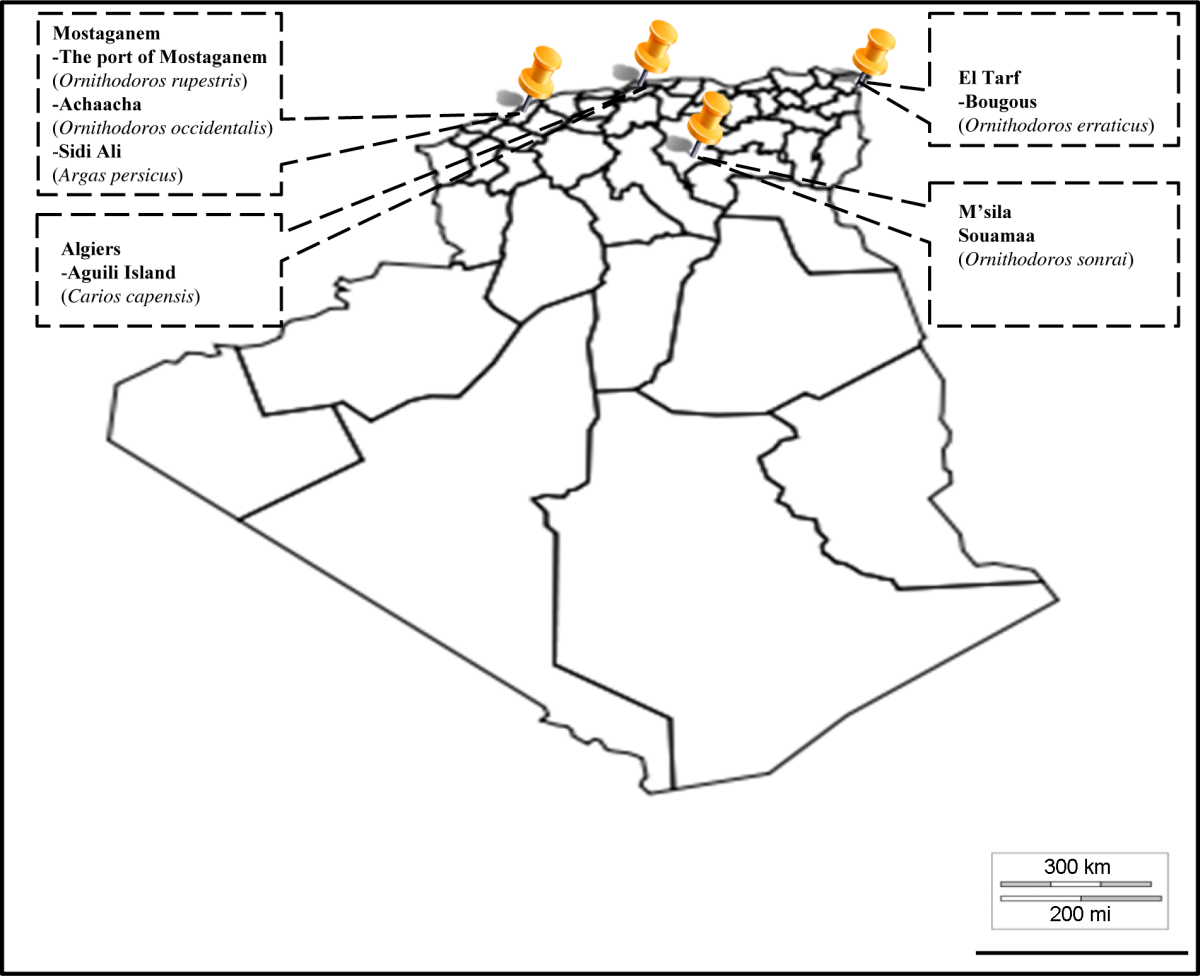
Geographical distribution of argasid ticks collected and screened for the presence of *Borrelia*, *Bartonella* and Anaplasmataceae DNA in Algeria.

In this study, 20 rodent burrows, 10 yellow-legged gull (*Larus michahellis*) nests and three animal shelters were prospectively sampled. A total of 204 ticks were collected at six sites. The identification of ticks using entomological keys and molecular tools is reported in Table 1. Six distinct species belonging to three genera were identified, including *Carios capensis*, *Ornithodoros rupestris*, *Ornithodoros occidentalis*, *Ornithodoros erraticus*, *Ornithodoros sonrai* and *Argas persicus* (Fig 1).

### Detection of *Borrelia* spp.

**Fig 2:**
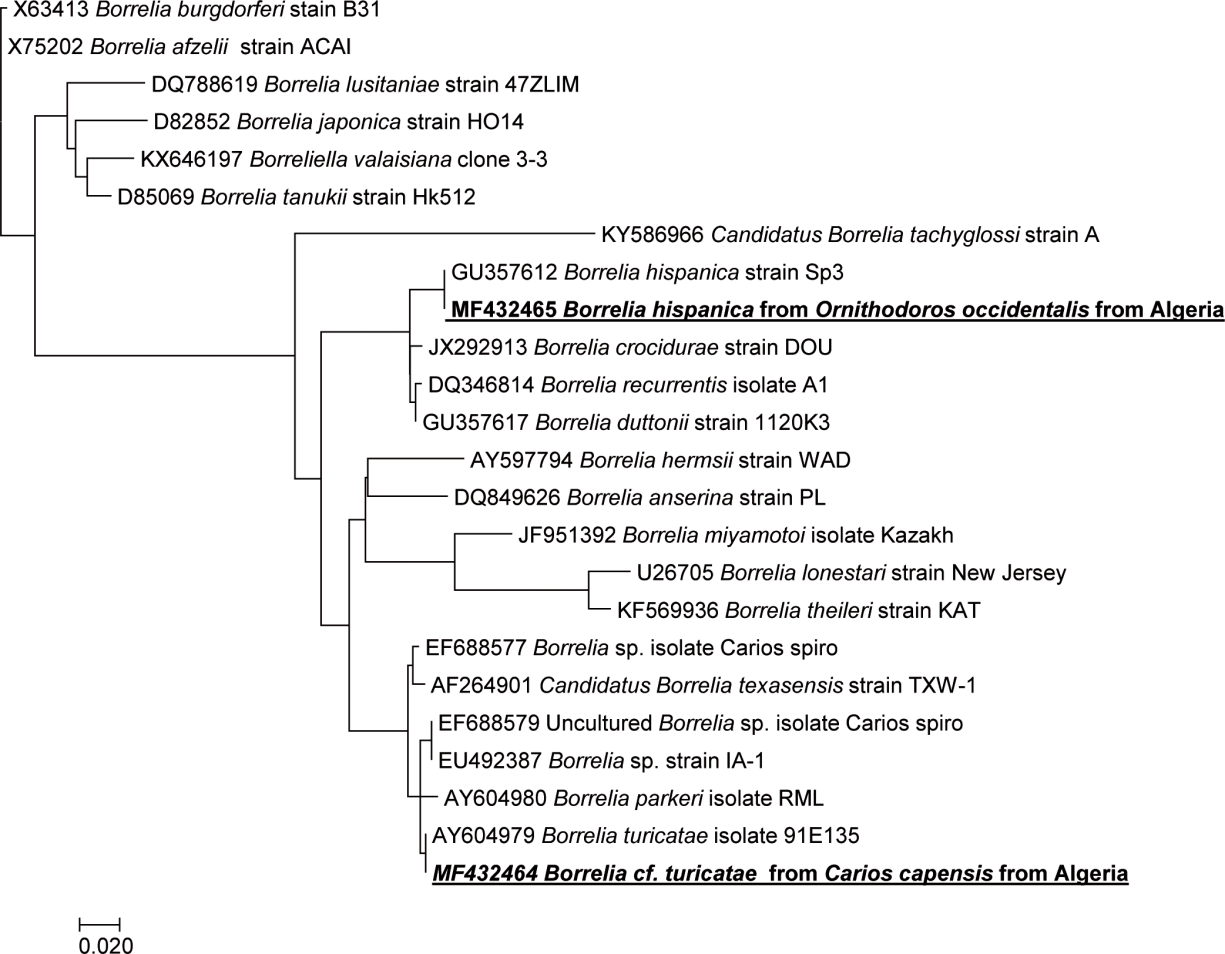
Phylogenetic tree showing the position of *Borrelia hispanica* amplified from *Ornithodoros occidentalis* and *Borrelia cf*. *turicatae* amplified from *Carios capensis* in this study compared to other species. Phylogenetic inferences were obtained using MEGA 6. GenBank accession numbers are indicated at the beginning and the geographic origin of the species is indicated at the end. The *flaB* gene of *Borrelia* sp sequences was aligned using CLUSTALW, and phylogenetic inferences obtained using the ML phylogenetic analysis with TOPALi 2.5 software (Biomathematics and Statistics Scotland, Edinburgh, UK) within the integrated ML application, using the K81uf + I + Г substitution model.

In Algiers, 5/48 (10.4%) of the *C*. *capensis* collected in *Larus michahellis* nests tested positive for *Borrelia* spp. by qPCR. Analysis of the sequenced 432 bps-long portion of the flagellin gene (*flaB*) showed 100% identity with several North American genotypes of *B*. *turicatae* (GenBank accession numbers CP015629, CP000049, AY604979 etc.) and 99.77% identity with another *B*. *turicatae* genotype (AY934630). The genotype identified here also showed a close identity with non-cultured *Borrelia spp*., including *Borrelia* sp. IA-1 from *Carios kelleyi* (EU492387) (98.84%), *Borrelia* sp. “Carios spiro-1” detected in a bat tick (EF688579) (98.84%), *Borrelia* sp. “Carios spiro-2” detected in a bat tick (EF688577) (98.56%), and *Borrelia parkeri* (AY604980) (98.84%) (Fig 2).

The ticks collected from the farm in Achaacha which were identified as *O*. *occidentalis* were 2/6 (33.3%) positive for *Borrelia* spp. by qPCR. DNA sequence analysis of the PCR products targeting the *flaB* gene showed 100% identity with *Borrelia hispanica* (Table 1).

All soft ticks which were screened from the port of Mostaganem, Sidi Ali (Douar Chtaouna), El Tarf and M’sila (34 *O*. *rupestris*, 50 *A*. *persicus*, 58 *O*. *erraticus* and 8 *O*. *sonrai*, respectively) were negative for *Borrelia* spp. by qPCR.

### Detection of *Bartonella* spp., *Rickettsia* spp. and Anaplasmataceae bacteria

**Fig 3:**
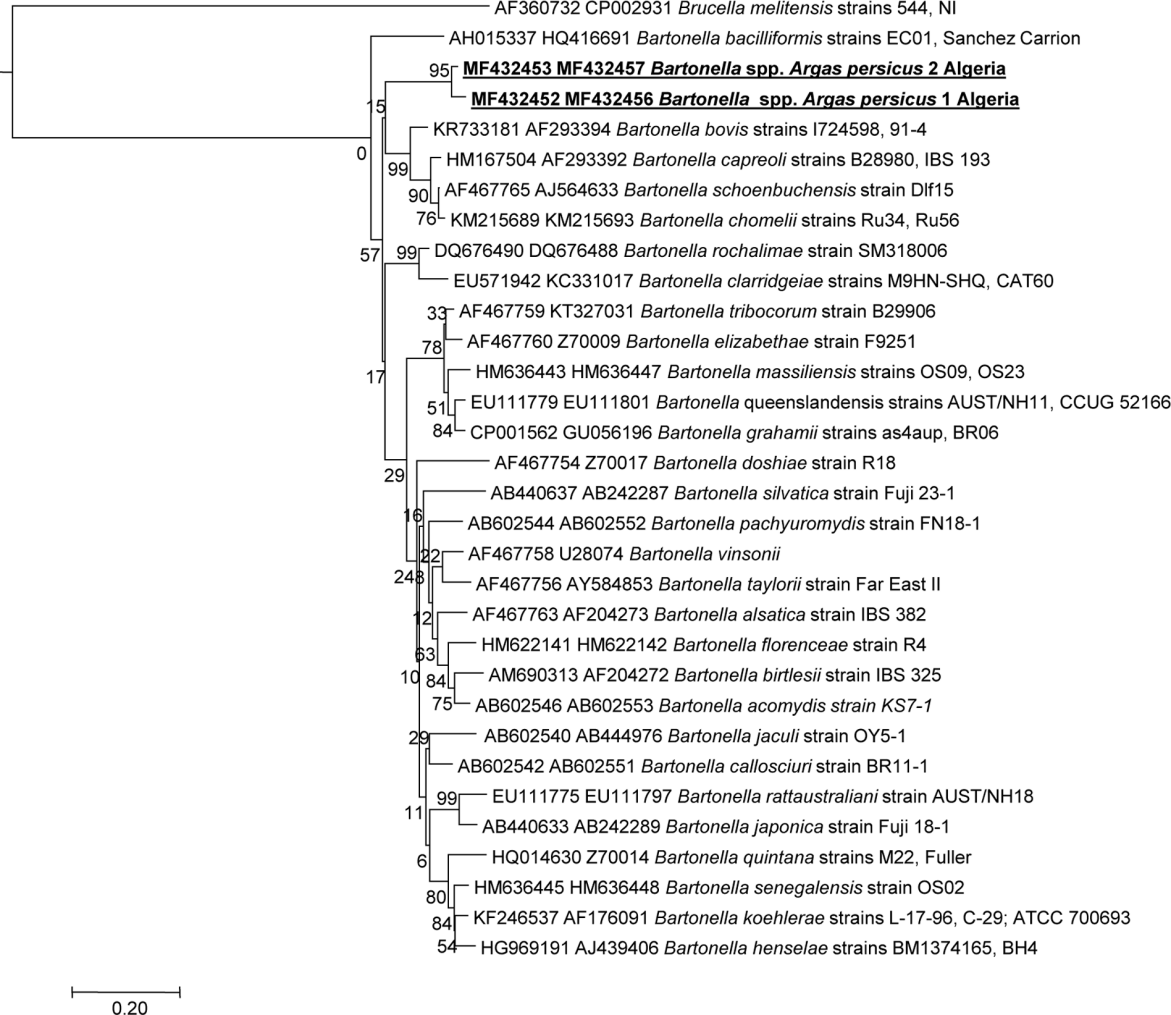
A consensus phylogenetic tree showing the relationships between *Bartonella* identified in the present study compared with other *Bartonella* species, based on concatenated *ftsZ* (292 bps) and *gltA* (200 bps) gene sequence comparisons. GenBank accession numbers are indicated at the beginning. Sequences were aligned using CLUSTALW, and phylogenetic inferences were obtained using the ML phylogenetic analysis with TOPALi 2.5 software (Biomathematics and Statistics Scotland, Edinburgh, UK) within the integrated ML application, using the K81uf + I + Г substitution model. Numbers at the nodes are percentages of bootstrap values obtained by repeating the analysis 100 times to generate a majority consensus tree (only those equal or higher than 80 were retained). The final set includes 492 base pairs. The scale bar represents a 20% nucleotide sequence divergence. Genbank accession numbers of *ftsZ* and *gltA* genes respectively are indicated at the beginning.

By qPCR, 31/50 (62%) *Argas persicus* ticks collected in Sidi Ali (Mostaganem) tested positive for *Anaplasmataceae* bacteria, 3/50 (6%) ticks tested positive for *Rickettsia* spp., and 4/50 (8%) ticks tested positive for *Bartonella* spp. These four ticks tested positive by standard PCR using primers targeting the *ftsZ* gene, and two were positive by PCR using *gltA* gene primers. Using DNA sequencing, we identified four *ftsZ* and two *gltA* sequences of *Bartonella* spp. (Table 1). The closest sequence available in GenBank was that of *Bartonella elizabethae* (AF467760; 96% similarity) from the American Type Culture Collection, Manassas VA, USA (F9251T/ATCC. 49927). A phylogenetic tree based on concatenated *ftsZ* (292 bps) and *gltA* (200 bps) genes showed that our genotype belongs to a cluster of *Bartonella* spp. including *Bartonella bovis*, *Bartonella capreoli*, *Bartonella schoenbuchensis* and *Bartonella chomelii* (Fig 3). Furthermore, standard PCR revealed that 17 samples tested positive for Anaplasmataceae DNA (23S rRNA gene). We also identified six DNA sequences of Anaplasmataceae bacteria in six *Argas persicus* ticks from Mostaganem (Table 1). The closest sequence available in GenBank was the sequence of *Anaplasma ovis* (KM021411.1; 93% similarity), isolated from sheep in Senegal. Although one sample tested positive for *Rickettsia* DNA (the *gltA* gene), the quality of the sequence obtained was too poor to be interpreted.

## Discussion

This investigation reports direct evidence of DNA from *Borrelia* spp., *Bartonella* spp., and an *Anaplasmataceae* bacterium in argasid ticks in Algeria.

In this study, we identified *Borrelia* DNA that, based on analysis of a portion of the *flaB* gene, is identical to *B*. *turicatae*. Few studies have been performed and little molecular data exists for *B*. *turicatae*, related species and isolates [4]. *Borrelia turicatae* is known as a New World agent of tick-borne relapsing fever, which is prevalent throughout the southern United States and Latin America, and maintained there in enzootic cycles by a soft tick, *O*. *turicata* [19]. This tick is present throughout Mexico and Central and South America, where it colonizes peridomestic settings and inhabits burrows, nests, caves, and cavities under rocky outcrops. *O*. *turicata* are also promiscuous feeders, and recognized hosts include prairie dogs, ground squirrels, snakes, cattle, pigs, and the gopher tortoise [19]. *Borrelia turicatae* is genetically closely related to *B*. *parkeri*, another New World agent of tick-borne relapsing fever. However, *B*. *parkeri* differs from both *B*. *turicatae* and *B*. *hermsii*, a causative agent of tick-borne relapsing fever in the western United States, by the lack of circular plasmids in its genome [20].

Our results, reporting the infection of *C*. *capensis* collected from Algerian seabird nests with *Borrelia turicatae*, might be considered surprising, as they do not support the tick-spirochete species specificity that has long been highlighted by previous authors. *C*. *capensis* are typically nest-associated ticks with a strong specificity for seabirds. They are known to infest the nests of the yellow-legged gull (*Larus michahellis*) along the coasts of Algeria [21]. However, our results are supported by the analysis of a portion of the *flaB* gene only. Borrelial flagellin is encoded by the *flaB* gene, which is genus-specific and highly conservative among *Borrelia* spp. Interestingly, the sequences of the flagellin gene may be identical in *Borrelia* spp. although they have different tick hosts, different epidemiology, and profound genomic differences (i.e., *B*. *recurrentis*, the agent of louse-borne relapsing fever, and *B*. *duttonii*, the agent of East-African tick-borne relapsing fever) [6,22]. However, due to a lack of DNA, we were unable to analyze more genes of the *Borrelia* spp. in this study. This is why we reference the genotype identified in this work as originating from *Borrelia cf*. *turicatae*.

Moreover, several *Borrelia* sequences which are very closely related to *Borrelia turicatae* sequences were recently reported from bat ticks in the USA [23]. An almost identical bacterium was also once found in another seagull-associated soft tick, *Carios sawaii* in Japan [24], as well as in African penguins [25]. Hence, it would appear that several *Borrelia* spp. closely related to *B*. *turicatae* are associated with seabirds and their ticks [25]. Our results offer additional evidence of this association and the consequences deserve further investigation. *Carios capensis* may also pose a threat to humans visiting yellow-legged gull rookeries if visitors are exposed to ticks harboring infectious agents.

*Borrelia hispanica* is an agent of tick-borne relapsing fever in the Old World. It is known to be transmitted to humans by infected *O*. *erraticus* ticks in Spain, Portugal, and Morocco [26]. In the Iberian Peninsula, this tick is associated with swine and rodents and their surroundings, where ticks remain buried in soil or crevices. Recently, *Borrelia hispanica* was found in *O*. *marocanus* (5/43, 11.6%) and *O*. *occidentalis* (3/55, 5.5%) in Morocco, and *O*. *kairouanensis* (1/5, 20.0%) in Tunisia [5]. In this study, *Borrelia hispanica* was detected for the first time in Algeria in *O*. *occidentalis* (2/6, 33.3%) collected from a farm owned by a patient with a previously unexplained fever in Achaacha (Mostaganem). Recently, *O*. *occidentalis* has been reported to belong, alongside *O*. *marocanus* and *O*. *kairouanensis*, to a complex of three large species (female adult length 5.5–7.5 mm) distributed in typically Mediterranean areas of Morocco, Algeria, Tunisia and Spain and previously confused with *O*. *erraticus* [12]. Interestingly, it has been reported that in northwestern Morocco, 20.5% of patients presenting with an unexplained fever had tick-borne relapsing fever related to *Borrelia hispanica* [27]. However, further epidemiological studies are recommended to screen patients with unexplained fever in Algeria.

Human illnesses associated with Bartonella occur worldwide, and they encompass a broad clinical spectrum, including fever, skin lesions, endocarditis, lymphadenopathy, and abnormalities of the central nervous system, eye, liver and bone [28]. Bartonelloses are infectious diseases caused by bacteria from the genus *Bartonella* which infects erythrocytes and endothelial cells in humans [29]. Biting arthropod vectors transmit these pathogens and infect a wide range of wild and domestic mammals, including rodents, cats, dogs, and cattle. Throughout our investigation, we identified *A*. *persicus Bartonella* spp. in 2/50 (4%) fowl ticks by sequence analysis of *ftsZ* and *gltA*. The phylogenetic tree demonstrates that our genotype belongs to the cluster of the *Bartonella* spp. family, including *Bartonella bovis* transmitted by *Haemaphysalis bispinosa* [30], *Bartonella capreoli* transmitted by *Ixodes ricinus* [31], *Bartonella schoenbuchensis* transmitted by *Melophagus ovinus*, and *Lipoptena cervi* for *Bartonella chomelii* [32].

*Argas persicus*, currently considered native to Turanian-Central Asia, is a parasite of arboreal nesting birds, and has successfully adapted to coexist with domestic fowl. Probably as the result of human transport, it has spread worldwide, where it survives practically exclusively in association with domestic fowl [33]. Its bite is painful to humans, often with toxic after-effects, but such episodes in humans are rare [34]. The fowl tick has been morphologically described previously in the Mediterranean basin and in Algeria [13]. It may transmit avian encephalomyelitis, leucocytozoonosis [35], and West Nile virus [36]. *A*. *persicus* could harbor different types of bacteria, including those of the genus *Salmonella*, *Aerobacter*, *Escherichia*, *Proteus*, *Staphylococcus*, *Flavobacterium*, *Bacillus*, *Pseudomonas*, and *Streptococcus* [37]. Here, the association between *Bartonella* sp. and *A*. *persicus* is reported for the first time in Algeria. Since ticks have been collected from animals that, in some cases, could be bacteremic, the ticks may be vectors for some pathogens, but also only carriers in other cases. Consequently, we cannot consider the presence of bacteria in an arthropod as proof of vector competence.

**Fig 4:**
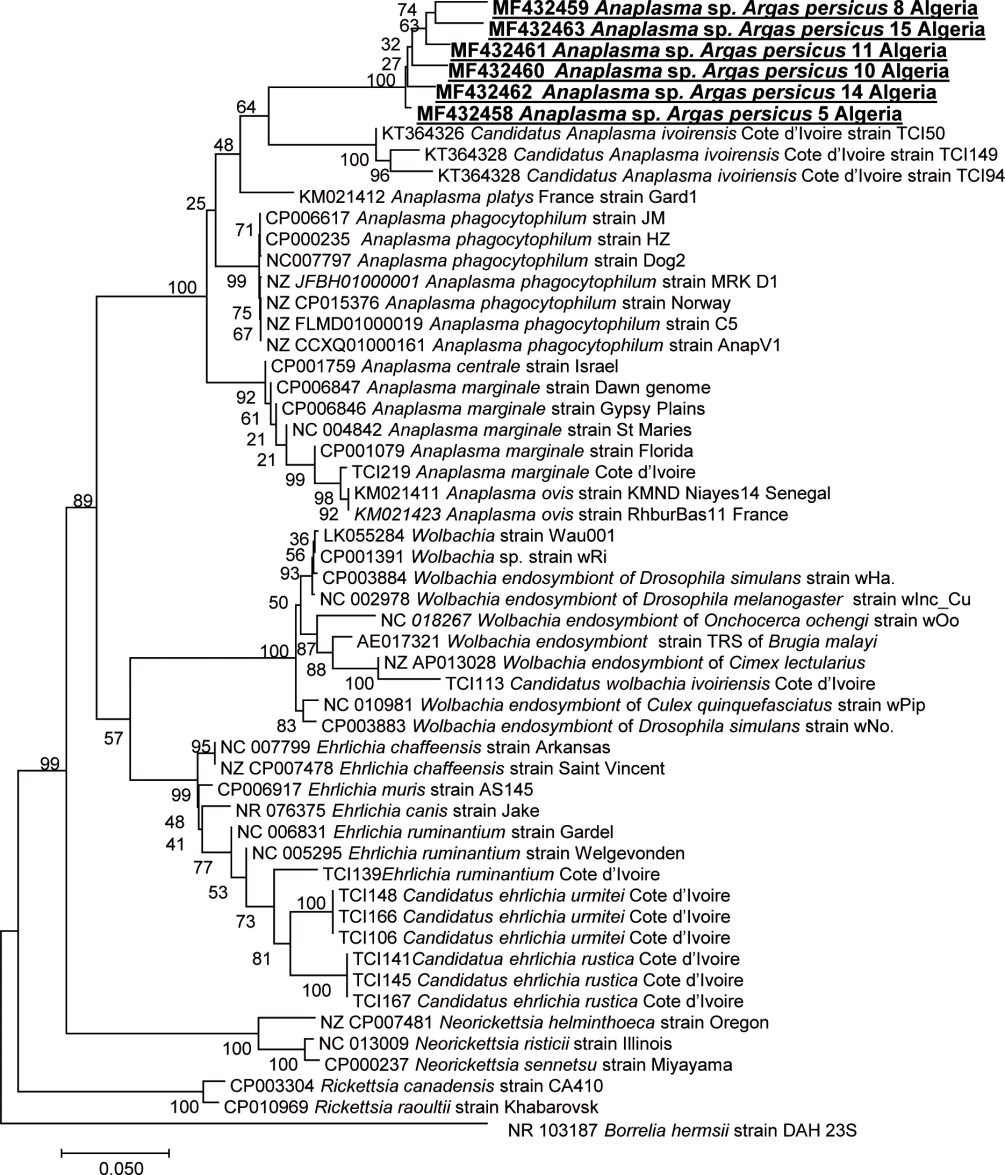
Phylogenetic tree showing the position of *Anaplasma* sp. amplified from *Argas persicus* in this study compared to other species. Phylogenetic inferences were obtained using MEGA 6. GenBank accession numbers are indicated at the beginning and the geographic origin of the species is indicated at the end. The 23S rRNA gene sequences were aligned using CLUSTALW, and phylogenetic inferences were obtained using the ML phylogenetic analysis with TOPALi 2.5 software (Biomathematics and Statistics Scotland, Edinburgh, UK) within the integrated ML application, using the K81uf + I + Г substitution model.

*Anaplasma* spp. are globally-distributed tick-transmitted bacteria of importance to veterinary and public health. These pathogens cause anaplasmosis in domestic and wild animal species, as well as in humans. *Rhipicephalus*, *Ixodes*, *Dermacentor* and *Amblyomma* genera of hard ticks include vectors of *Anaplasma* spp. [38]. Here, we identified a new genotype of *Anaplasmataceae* bacteria in 6/50 *Argas persicus*. In the phylogenetic tree, *Anaplasma* amplified from ticks collected in this study were closely related to “*Candidatus Anaplasma ivoirensis”* (KT364326) detected in *Amblyomma variegatum* ticks in Cote d'Ivoire, *Anaplasma ovis* strain KMND (KM021411) detected in sheep in Senegal [39], and *Anaplasma marginale* (TCI219) detected from *Rhipicephalus microplus* in Cote d’Ivoire (Fig 4). The pathogenicity of the *Anaplasma* genotype detected in our study is still unknown, although the closely related *Anaplasma marginale* is a globally-distributed tick-borne disease agent with great economic importance in the world’s cattle industry [40]. Interestingly, in vitro cultivation of *A*. *marginale* and *A*. *phagocytophilum* in argasid ticks cell lines has been achieved [41]. As discussed above, the detection of DNA in a tick does not mean that this tick is an efficient vector of the bacteria. There is a lack of information about *Anaplasmataceae* infection in argasid ticks, and our findings represent the first report of argasid tick infection with *Anaplasma* in Algeria. Unfortunately, due to the lack of DNA, we were unable to amplify and sequence other gene fragments in order to better characterize our Anaplasmataceae genotypes as belonging to new species.

Finally, for the first time in Algeria, we detected *Borrelia cf*. *turicatae* in *C*. *capensis*, and *Borrelia hispanica* in *O*. *occidentalis*, in two coastal regions of Algeria. We also detected one genus of *Bartonella* spp. and one genus of Anaplasmataceae bacteria in *A*. *persicus*. We have expanded the understanding of the repertoire of argasid ticks with the first molecular evidence of *A*. *persicus* in Mostaganem and tick-borne bacteria present in these arthropods in Algeria. Our findings will help human and veterinary clinicians to enlarge the spectrum of pathogens to be considered in differential diagnosis. Further work is needed to map tick/borrelia distribution in relation to environmental and climatic characteristics.
